# Supplementary material for: Development and testing of a polygenic risk score for breast cancer aggressiveness
Source: NPJ Precis Oncol. 2023 May 15;7:42. doi: 10.1038/s41698-023-00382-z (PMC10185660; doi:10.1038/s41698-023-00382-z)

Development and testing of a polygenic risk score for breast cancer aggressiveness

Supplementary Materials

Supplementary Table 1. Datasets used in the development of the polygenic risk score for risk of recurrence score weighted on proliferation (ROR-P PRS)

Study	Number of cases	Selection criteria for cases	Gene expression profiling method	SNP genotyping platform	Imputation reference	Quality control
The Cancer Genome Atlas (TCGA)	953	Invasive breast cancers, recurrences excluded	RNA-Seq	Affymetrix SNP 6.0	1000 Genomes	SNPs excluded if minor allele frequency <0.5% or variant missing rate >5%. Individuals excluded if missing call rate > 5%.
Molecular Taxonomy of Breast Cancer International Consortium (METABRIC)	496	Genotyped subset	Illumina HT-12 beadchip	Affymetrix SNP 6.0	1000 Genomes	
Investigation of Serial Studies to Predict Your Therapeutic Response with Imaging And molecular analysis 2 (I-SPY 2 TRIAL)	914	First 1400 consecutive cases underwent genotyping; ROR-P available for a subset	Agilent 44k array	Affymetrix Axiom Precision Medicine Diversity Array	1000 Genomes	

Supplementary Table 2. Datasets used to test the association between the polygenic risk score for the risk of recurrence score weighted on proliferation (ROR-P PRS) with clinical outcomes

Study	Cases	Breast cancer deaths	Years of diagnosis, cases	Length of follow-up, median (IQR)	SNP genotyping platform	Imputation reference	Quality control
UK Biobank	7,427	544	2006-2019	6.4 years	Affymetrix UK Biobank Axiom Array	Haplotype Reference Consortium (primary); UK10K, 1000 Genomes (secondary)	SNPs excluded if: variant missing rate >3%; minor allele frequency <0.01; heterozygosity more than 5 standard deviations from mean
Pathways Study	2,769	241	2006-2013	10.7 years	Illumina MultiEthnic Global-8	Haplotype Reference Consortium	SNPs excluded if: variant missing rate >2%; >1 Mendelian error in the HapMap trios included in genotyping; Hardy–Weinberg equilibrium ($p < 1\times10^{-4}$); discordance between duplicate samples; or positional duplicated variants.

Supplementary Table 3. Characteristics of studies used for survival analysis of the polygenic risk score for the risk of recurrence score weighted on proliferation (ROR-P PRS)

Characteristic	UK Biobank<br>N = 7,427[1]	Pathways Study<br>N = 2,769[1]
Age at diagnosis (years)	64 (57, 69)	62 (54, 70)
Breast cancer deaths	544 (7.3%)	241 (8.7%)
Follow-up time (years)	6.4 (3.7, 9.1)	10.7 (8.2, 12.3)
Body mass index		27 (24, 32)
Unknown		1
Estrogen receptor status		
positive		2,386 (86%)
negative		382 (14%)
Unknown		1
HER2 status		
positive		319 (12%)
negative		2,337 (88%)
Unknown		113
Histologic grade		
1		795 (31%)
2		1,192 (46%)
3		612 (24%)
Unknown		170
IHC subtype		
Luminal A		2,066 (78%)
Luminal B		224 (8.4%)
Basal		270 (10%)
HER2		95 (3.6%)
Unknown		114
ROR-P		45 (17, 63)
Unknown		2,285

	UK Biobank	Pathways Study
Characteristic	N = 7,427[1]	N = 2,769[1]
ROR-P Group		
Low		96 (20%)
Medium		194 (40%)
High		194 (40%)
Unknown		2,285

[1]Median (IQR); n (%)

HER2, human epidermal growth factor receptor 2; IQR, interquartile range; ROR-P, risk of recurrence score weighted on proliferation

Supplementary Table 4. Candidate single nucleotide polymorphisms for polygenic risk score for the risk of recurrence score weighted on proliferation (ROR-P PRS)

See separate Excel file.

Supplementary Table 5. Performance of polygenic risk scores for the risk of recurrence score weighted on proliferation (ROR-P PRS) by p-value threshold

p-value threshold	Number of SNPs	RMSE	R-squared	MAE
0.6	124	21.78	0.0382	17.74
0.575	119	21.73	0.0400	17.70
0.55	111	21.68	0.0418	17.66
0.525	107	21.68	0.0411	17.65
0.5	104	21.65	0.0419	17.63
0.475	98	21.61	0.0434	17.60
0.45	92	21.58	0.0449	17.57
0.425	91	21.57	0.0450	17.57
0.4	84	21.52	0.0475	17.51
0.375	76	21.47	0.0489	17.49
0.35	67	21.54	0.0419	17.53
0.325	62	21.52	0.0420	17.52
0.3	59	21.65	0.0315	17.66
0.275	54	21.63	0.0317	17.64
0.25	50	21.60	0.0330	17.62
0.225	48	21.59	0.0331	17.62
0.2	42	21.60	0.0315	17.62
0.175	34	21.61	0.0298	17.64
0.15	28	21.64	0.0254	17.65
0.125	23	21.65	0.0233	17.66
0.1	19	21.68	0.0199	17.69

Supplementary Table 6. Associations between single nucleotide polymorphisms (SNPs) and the risk of recurrence score weighted on proliferation (ROR-P) for the 76-SNP ROR-P polygenic risk score

Variant	chr	position	Mapped Gene(s)	risk	other	Coefficient	p-value
rs2992756	1	18807339	IGSF21, KLHDC7A	T	C	-0.566	0.354
rs1707302	1	46600917	P3R3URF-PIK3R3	G	A	-0.651	0.294
rs12118297	1	87779217	LMO4, LINC02801	T	G	1.171	0.130
rs4971059	1	155148781	TRIM46	A	G	-1.143	0.069
rs4951011	1	203766331	ZBED6, ZC3H11A	G	A	0.719	0.357
rs4245739	1	204518842	MDM4	C	A	1.092	0.112
rs11117758	1	217220574	ESRRG	G	A	-0.740	0.333
rs71801447	2	111925731	MIR4435-2HG, BCL2L11	C	CTTATGTT	1.035	0.348
rs4442975	2	217920769	LINC01921, RN7SKP43, AC007749.1, AC007749.2	G	T	-0.905	0.138
rs34005590	2	217963060	LINC01921, RN7SKP43	C	A	-2.868	0.056
rs12479355	2	227226952	NYAP2, MIR5702	A	G	-0.775	0.296
rs6762644	3	4742276	ITPR1	G	A	-0.696	0.275
rs1375631	3	16778867	CDYLP1, PLCL2	G	A	-0.853	0.176
rs12493607	3	30682939	TGFBR2	C	G	-0.947	0.151
rs1053338	3	63967900	ATXN7	G	A	-1.297	0.164
rs13066793	3	87037543	VGLL3	A	G	-1.509	0.215
rs9833888	3	99723580	CMSS1, FILIP1L	T	G	-1.202	0.101
rs58058861	3	172285237	LINC02068	A	G	0.953	0.202
rs495367	4	1986972	NELFA	G	A	0.868	0.176
rs10022462	4	89243818	PPM1K-DT	T	C	-0.928	0.136
rs72749841	5	49641645	NA, EMB	T	C	-1.439	0.305
rs62355902	5	56053723	C5orf67, MAP3K1, AC008940.1	T	A	-0.822	0.302
rs1353747	5	58337481	PDE4D	T	G	1.605	0.149
rs138044103	5	67424121	NA, RNU6-1232P	C	CTG	-0.652	0.282
rs421379	5	91275313	AC114316.1, PCBP2P3	T	C	1.387	0.188
rs6882649	5	111217786	NREP	T	G	-1.118	0.082
rs11242675	6	1318878	FOXQ1, LINC01394	T	C	-1.237	0.043
rs71557345	6	26680698	ZNF322, NA	G	A	2.482	0.159
rs9383951	6	152295613	ESR1	C	G	-2.525	0.204
rs2747652	6	152437016	ESR1, AL078582.1	C	T	-0.990	0.098
rs188092014	7	74341926	SPDYE12P, CASTOR2	C	G	1.128	0.275
rs71559437	7	101552440	CUX1	G	A	1.344	0.186
rs720475	7	144074929	ARHGEF5	G	A	-0.769	0.292
rs66823261	8	170692	RPL23AP53	C	T	-0.683	0.349

rs142890050	8	23480253	RNU4-71P, SINHCAFP3	C	CTT	1.584	0.008
rs13365225	8	36858483	KCNU1, SMARCE1P4, AC092818.1, AC090453.1	A	G	-0.740	0.321
rs58847541	8	124610166	RN7SKP155, KLHL38	A	G	-1.406	0.081
rs17350191	8	124757661	ANXA13, FAM91A1	T	C	0.844	0.191
rs4742903	9	106856793	SMC2	C	G	0.900	0.152
rs10760444	9	129396434	LMX1B	G	A	-0.913	0.127
rs2380205	10	5886734	ANKRD16, GDI2	C	T	0.606	0.327
rs7072776	10	22032942	MLLT10, DNAJC1	A	G	-0.768	0.235
rs140936696	10	95292187	CEP55, FFAR4	CAA	C	-0.939	0.209
rs11199914	10	123093901	LINC01153, RN7SKP167	C	T	-0.580	0.360
rs35054928	10	123340431	FGFR2	GC	G	-1.101	0.075
rs2912774	10	123348662	FGFR2	T	G	-1.114	0.071
rs45631563	10	123349324	FGFR2	A	T	-2.165	0.188
rs6597981	11	803017	PIDD1	G	A	0.615	0.331
rs537626	11	69307695	LINC01488	C	G	-2.169	0.008
rs2059614	11	125259424	PKNOX2	G	A	-2.502	0.044
rs7297051	12	28174817	PTHLH, CCDC91	C	T	1.413	0.054
rs1027113	12	29140260	NA	A	G	-1.396	0.218
rs202049448	12	85009437	RPL6P25, SLC6A15	T	C	-1.500	0.018
rs11065822	12	111600134	CUX2	G	T	-0.955	0.162
rs1061657	12	115108136	TBX3	C	T	-0.681	0.331
rs1292011	12	115836522	TBX3, UBA52P7, AC009803.2, AC078880.2	A	G	-1.128	0.063
rs206966	12	120832146	RPS27P25, COX6A1	T	C	1.114	0.174
rs3784099	14	68749927	RAD51B	A	G	-0.791	0.237
rs11076805	16	4106788	ADCY9	C	A	0.928	0.191
rs2432539	16	56420987	AMFR	A	G	0.933	0.138
rs4496150	16	87085237	LINC02188, LINC02181	C	A	1.153	0.104
rs72826962	17	40836389	CNTNAP1	T	C	-4.332	0.195
rs745570	17	77781725	CBX8, LINC01977	A	G	-1.062	0.086
rs1436904	18	24570667	CHST9, AQP4-AS1	T	G	-1.170	0.059
rs36194942	18	25401204	AC090936.1, CDH2	A	AT	0.995	0.125
rs67397200	19	17401404	ANKLE1, ABHD8	G	C	1.868	0.005
rs2965183	19	19545696	GATAD2A	A	G	0.727	0.248
rs113701136	19	30277729	AC008798.3, CCNE1	T	C	0.753	0.247
rs71338792	19	46183031	GIPR	AT	A	-0.859	0.289
rs141526427	20	11502618	NA, PGAM3P	A	AAC	-1.123	0.090
rs6065254	20	39248265	NA, MAFB	0	0	-1.082	0.082
rs6122906	20	48945911	LINC01271, RN7SL636P	G	A	-1.137	0.122

rs2823093	21	16520832	NRIP1, CYCSP42	G	A	-0.905	0.183
rs738321	22	38568833	PLA2G6	C	G	-0.560	0.358
rs6001930	22	40876234	MRTFA	C	T	-1.705	0.075
rs73161324	22	42038786	XRCC6	T	C	-2.739	0.091

Supplementary Table 7. Results of Cox proportional hazards models of the polygenic risk score for the risk of recurrence score weighted on proliferation (ROR-P PRS) versus breast cancer survival and invasive recurrence in the Pathways Study

	**Breast cancer death**		**Invasive recurrence**	
	HR (95% CI)	p-value	HR (95% CI)	p-value
**Model 1**[a]				
ROR-P PRS	1.14 (1.01-1.29)	4.1×10^{-2}	1.11 (1.00-1.24)	4.2×10^{-2}
**Model 2**[a]				
ROR-P PRS	1.14 (1.00-1.29)	4.5×10^{-2}	1.12 (1.01-1.24)	4.0×10^{-2}
Age at diagnosis	1.01 (1.00-1.02)	4.8×10^{-2}	1.00 (0.99-1.01)	4.0×10^{-1}
BMI	1.02 (1.00-1.04)	1.6×10^{-2}	1.01 (0.99-1.02)	3.5×10^{-1}
**Model 3**[a,b]				
ROR-P PRS	1.10 (0.97-1.25)	1.3×10^{-1}	1.11 (1.00-1.23)	5.6×10^{-2}
Age at diagnosis	1.02 (1.01-1.03)	2.4×10^{-3}	1.00 (0.99-1.01)	6.2×10^{-1}
BMI	1.02 (1.00-1.04)	6.6×10^{-2}	1.00 (0.99-1.02)	8.9×10^{-1}
Stage[c]				
I	Ref.		Ref.	
II	2.65 (1.88-3.73)	3.0×10^{-8}	2.30 (1.79-2.97)	1.2×10^{-10}
III	8.55 (5.94-12.3)	$<2 \times 10^{-16}$	5.44 (4.06-7.28)	$<2 \times 10^{-16}$
IV	44.5 (28.1-70.4)	$<2 \times 10^{-16}$	5.43 (3.04-9.69)	1.0×10^{-8}
**Model 4**[a,b]				
ROR-P PRS	1.09 (0.96-1.24)	1.8×10^{-1}	1.10 (0.99-1.22)	7.1×10^{-2}
Age at diagnosis	1.03 (1.02-1.04)	8.4×10^{-6}	1.01 (1.00-1.02)	2.7×10^{-1}
BMI	1.02 (1.00-1.04)	7.1×10^{-2}	1.00 (0.99-1.02)	8.6×10^{-1}
Stage[c]				
I	Ref.		Ref.	
II	1.95 (1.34-2.84)	5.5×10^{-4}	2.08 (1.57-2.75)	2.8×10^{-7}
III	5.90 (3.87-8.99)	$<2 \times 10^{-16}$	4.75 (3.39-6.64)	$<2 \times 10^{-16}$
IV	36.2 (22.4-58.4)	$<2 \times 10^{-16}$	4.38 (2.40-7.97)	1.4×10^{-6}
Chemotherapy	2.15 (1.32-3.50)	2.0×10^{-3}	1.26 (0.83-1.91)	2.8×10^{-1}
Taxane therapy	0.91 (0.57-1.45)	6.9×10^{-1}	0.98 (0.65-1.47)	9.1×10^{-1}
Hormonal therapy	0.50 (0.37-0.66)	1.2×10^{-6}	0.73 (0.58-0.91)	5.7×10^{-3}
Radiation therapy	0.74 (0.55-1.00)	5.1×10^{-2}	0.88 (0.69-1.12)	2.9×10^{-1}
Trastuzumab	0.53 (0.37-0.75)	4.5×10^{-4}	0.89 (0.68-1.18)	4.1×10^{-1}
**Model 5**[a, c]				
ROR-P PRS	0.95 (0.77-1.17)	6.2×10^{-1}	1.01 (0.83-1.22)	9.5×10^{-1}
ROR-P	1.02 (1.01-1.03)	9.6×10^{-4}	1.01 (1.00-1.02)	4.7×10^{-2}
**Model 6**[a]				
ROR-P PRS	1.12 (0.98-1.27)	8.7×10^{-2}	1.10 (0.99-1.23)	6.4×10^{-2}
ER status				
Positive	Ref.		Ref.	
Negative	2.48 (1.86-3.30)	7.2×10^{-10}	1.79 (1.38-2.32)	1.3×10^{-5}

[a]All models are adjusted for genetic ancestry, PC1-10
[b]Model includes subset of 2,689 cancers with available stage data with 230 breast cancer deaths
[c]American Joint Committee on Cancer (AJCC) Cancer Staging Manual, 6th Edition
[c]Model includes subset of 484 cancers with available ROR-P with 87 events
Abbreviations: BMI, body mass index; CI, confidence interval; ER, estrogen receptor; HR, hazard ratio; PRS, polygenic risk score; ROR-P, risk of recurrence score weighted on proliferation; Tx., therapy

Supplementary Table 8. Results of Cox proportional hazards models for the risk of recurrence score weighted on proliferation (ROR-P PRS), risk of estrogen-negative versus positive breast cancer ($PRS_{ER-/ER+}$), and joint model including both ROR-P PRS and $PRS_{ER-/ER+}$

	**UK Biobank**		**Pathways**		**Meta-analysis**				
	HR (95% CI)	p-value	HR (95% CI)	p-value	HR (95% CI)	p-value	Q	P_{het}	I^2
ROR-P PRS	1.13 (1.04-1.23)	$5.4x10^{-3}$	1.14 (1.01-1.29)	$4.1x10^{-2}$	1.13 (1.06-1.21)	$4.0x10^{-4}$	0.01	0.91	0%
$PRS_{ER-/ER+}$	1.15 (1.07-1.23)	$6.8x10^{-5}$	1.12 (1.00-1.26)	$5.7x10^{-2}$	1.14 (1.08-1.22)	$<1.0x10^{-4}$	0.15	0.70	0%
**Joint model**									
ROR-P PRS	1.09 (1.00-1.19)	$5.8x10^{-2}$	1.11 (0.97-1.27)	$1.2x10^{-1}$	1.10 (1.02-1.18)	$1.4x10^{-2}$	0.05	0.82	0%
$PRS_{ER-/ER+}$	1.13 (1.05-1.21)	$1.0x10^{-3}$	1.09 (0.96-1.23)	$2.0x10^{-1}$	1.12 (1.05-1.19)	$3.0x10^{-4}$	0.24	0.62	0%

All models are adjusted for genetic ancestry, PC1-10
Abbreviations: CI, confidence interval; HR, hazard ratio; PRS, polygenic risk score; ROR-P, risk of recurrence score weighted on proliferation

Supplementary Figure 1. Distribution of the risk of recurrence score weighted on proliferation (ROR-P) in the polygenic risk score (PRS) development datasets

Histograms of ROR-P in breast cancers from (A) The Cancer Genome Atlas, n=953; (B) Molecular Taxonomy of Breast Cancer International Consortium (METABRIC), n=496; and (C) Investigation of Serial Studies to Predict Your Therapeutic Response with Imaging And molecular analysis 2 (I-SPY 2 TRIAL), n=914.

A.

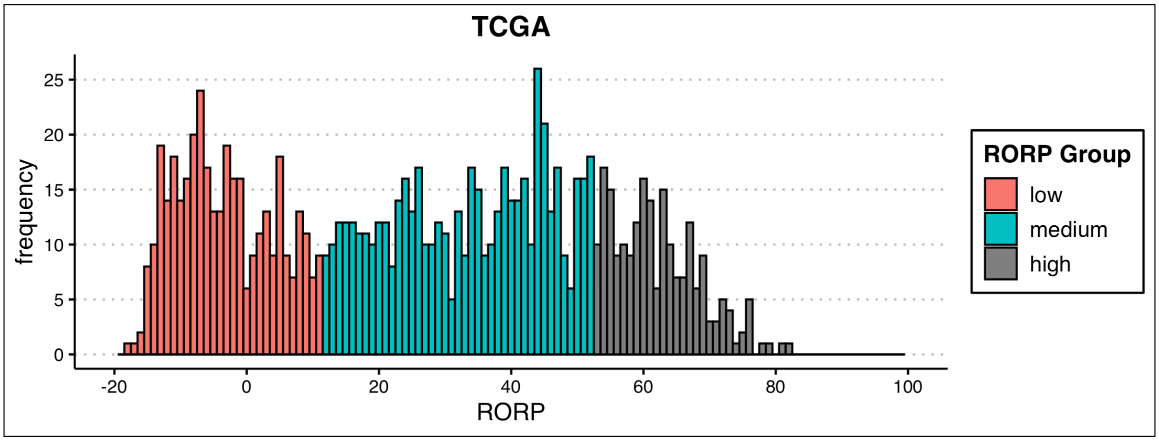

B.

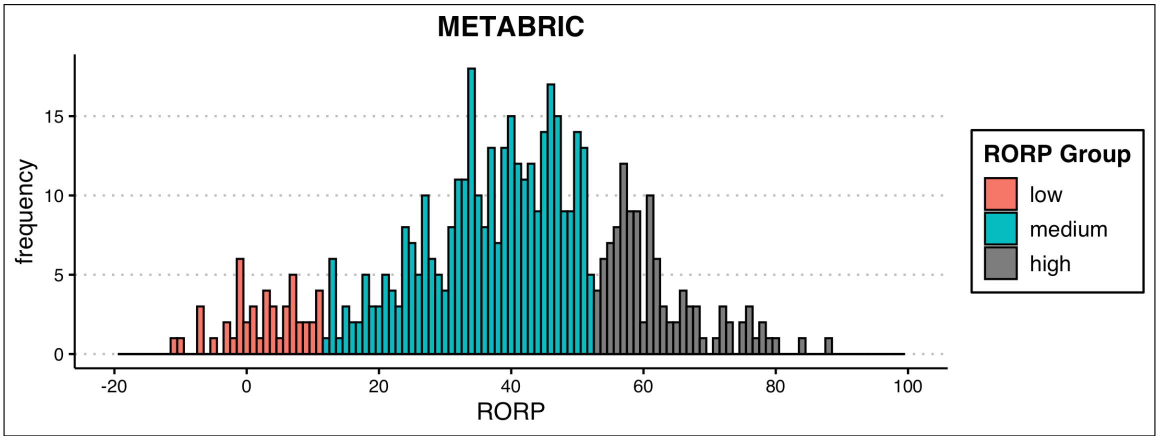

C.

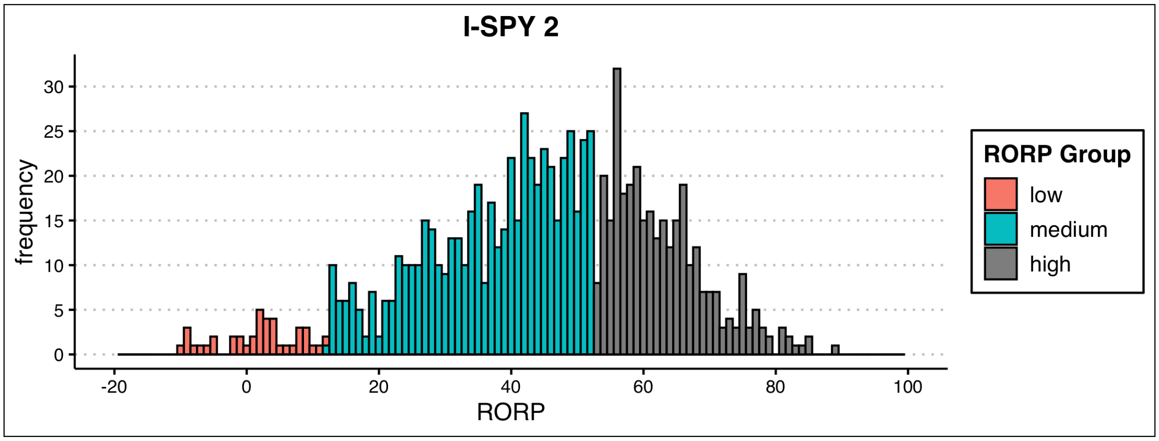

Supplementary Figure 2. Distribution of the risk of recurrence score weighted on proliferation (ROR-P) in the Pathways Study

Histogram of ROR-P breast cancers from the Pathways Study that underwent tumor gene expression profiling, n=484.

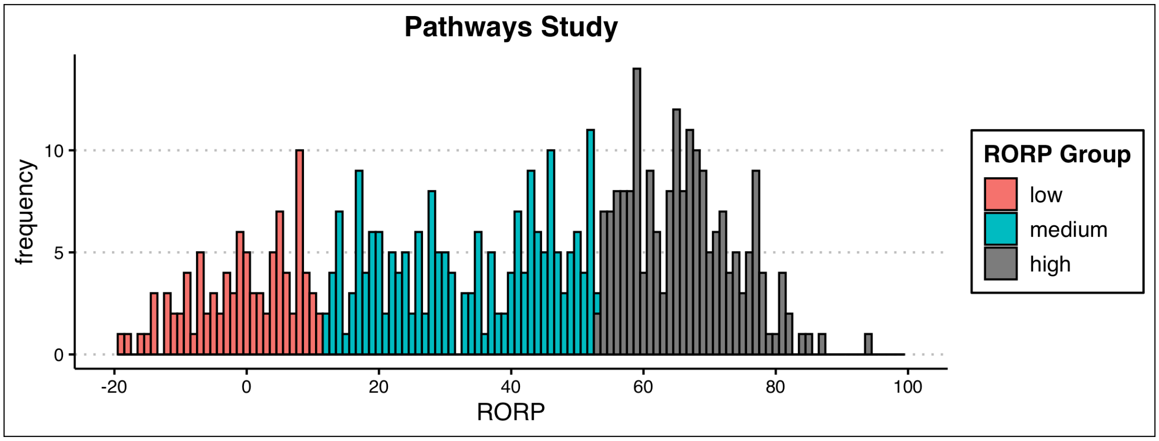

Supplementary Figure 3. Distributions of polygenic risk score for the risk of recurrence score weighted on proliferation (ROR-P PRS) in UK Biobank and the Pathways Study

Histograms of the ROR-P PRS PRS are shown for (A) UK Biobank, (B) the Pathways Study. The mean (standard deviation) of the ROR-P PRS was -28.2 (5.0) in the UK Biobank and -31.0 (5.0) in the Pathways Study.

A.

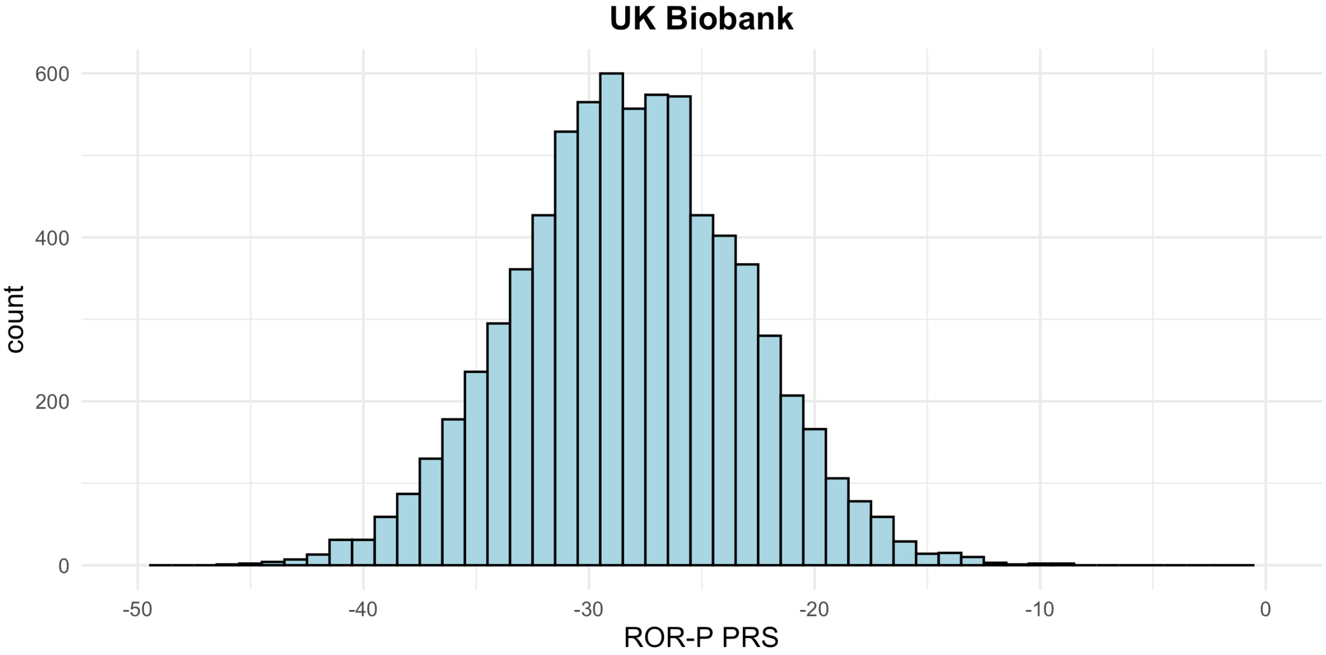

B.

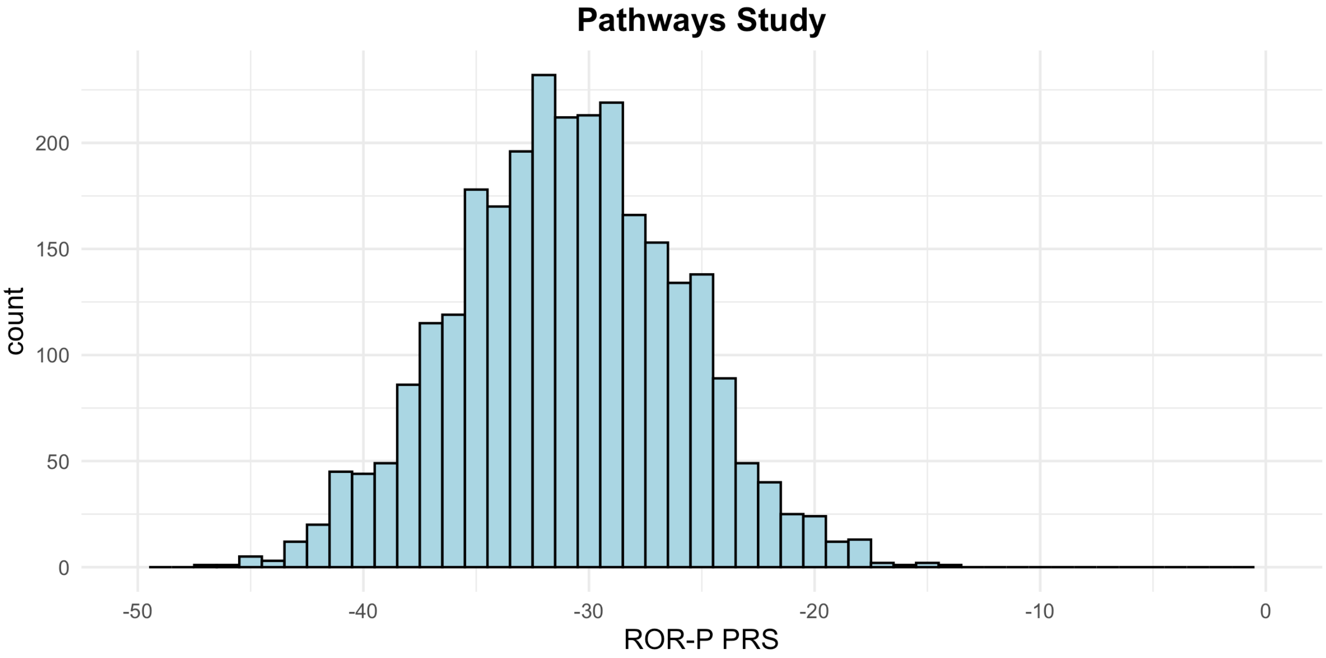

Supplementary Figure 4. Calibration of the polygenic risk score for the risk of recurrence score weighted on proliferation (ROR-P PRS) in the UK Biobank

Predicted versus observed 5-year survival based on the ROR-P PRS are plotted for incident cases in the UK Biobank. Estimates are shown for 7,427 participants divided into 10 strata of predicted survival probability. Black dots correspond to the Kaplan-Meier survival estimate (with 95% error bars) for each stratum of predicted survival. Blue X's correspond to bias-corrected estimates. The gray line corresponds to ideal calibration. The distribution of predicted survival probabilities is shown along the upper horizontal axis.

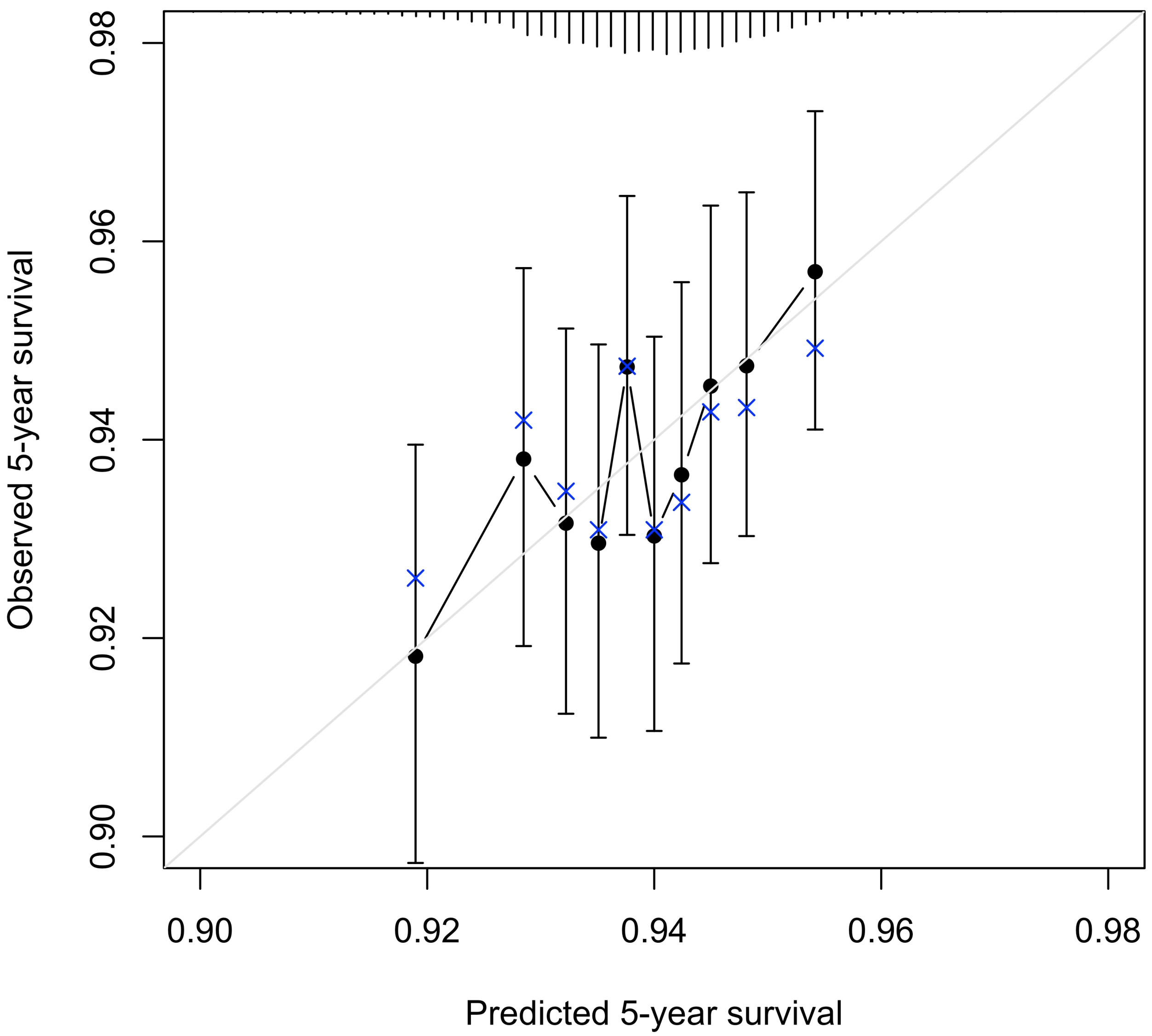

Supplementary Figure 5. Validation of the polygenic risk score for estrogen-negative versus positive breast cancer ($PRS_{ER-/ER+}$)

Boxplot of $PRS_{ER-/ER+}$ by immunohistochemical estrogen receptor status in Pathways. Boxes represent the interquartile range (IQR) and the center lines represent the median. Whiskers denote the minimum and maximum values, calculated as the first quartile – 1.5*IQR and third quartile + 1.5*IQR, respectively. Dots represent outliers beyond the minimum and maximum values. The p-value for the t-test is shown.

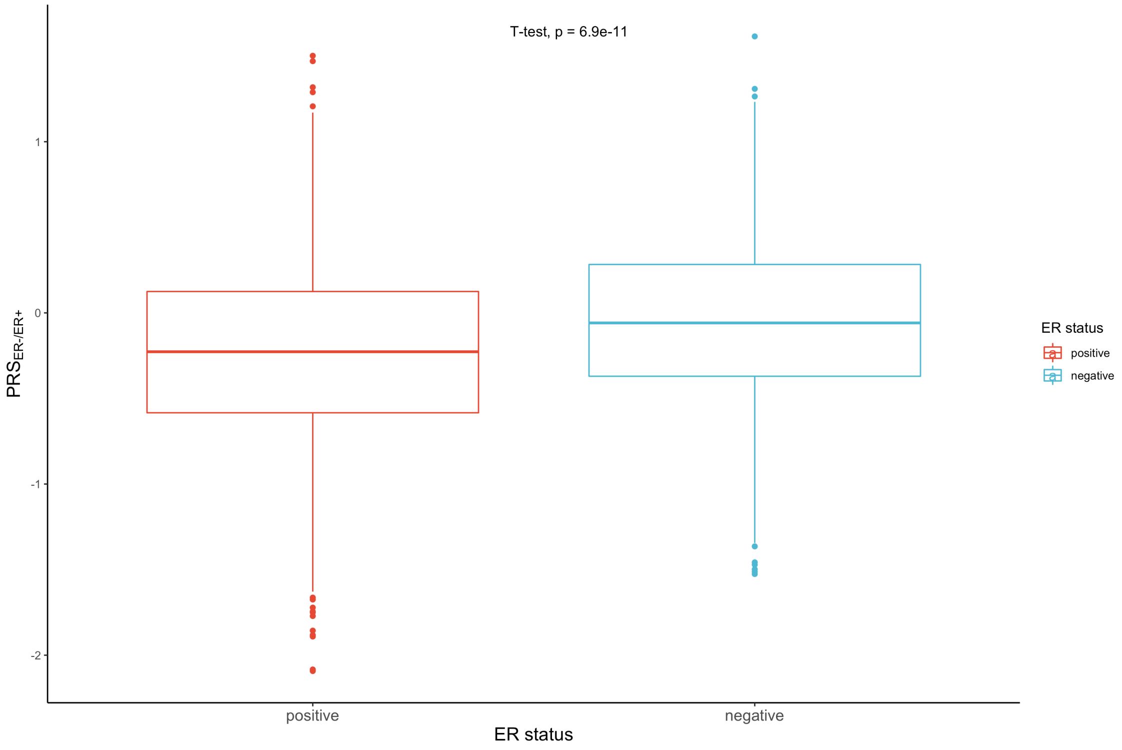

Supplementary Figure 6. Correlation between polygenic risk scores for the risk of recurrence score weighted on proliferation (ROR-P PRS) and risk of estrogen-negative versus positive breast cancer ($PRS_{ER-/ER+}$) in the UK Biobank and Pathways Study

Scatter plot of the normalized ROR-P PRS and log-transformed $PRS_{ER-/ER+}$ in the (A) UK Biobank and (B) the Pathways Study. The Pearson correlation coefficient and p-value are shown.

A.

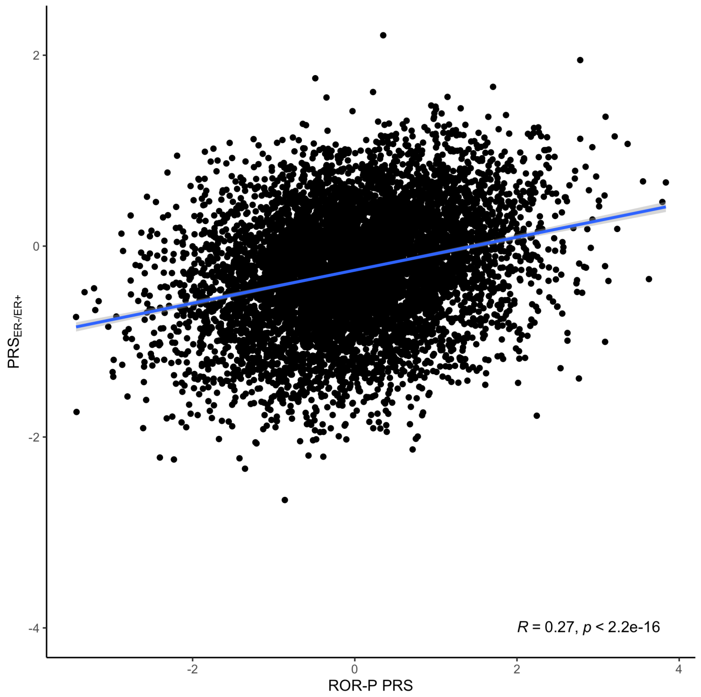

B.

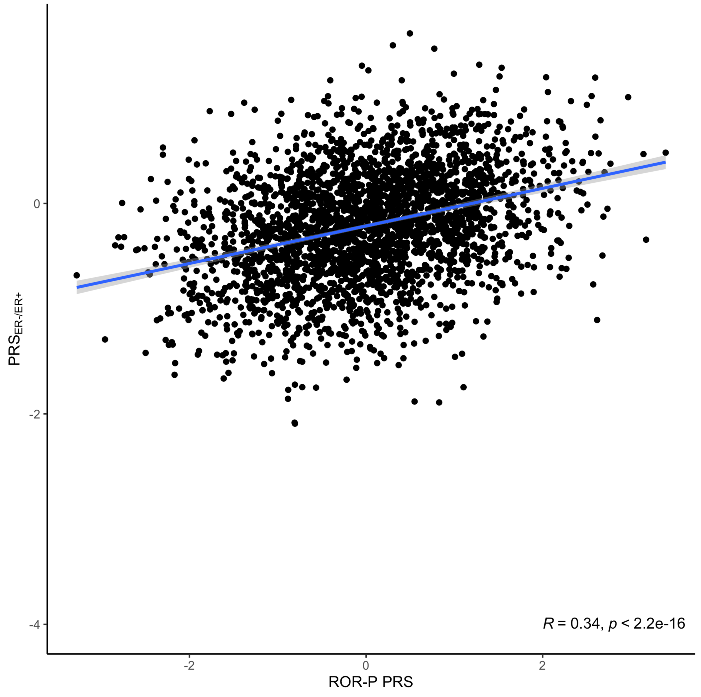

Supplementary Figure 7. Performance of the polygenic risk score for the risk of recurrence score weighted on proliferation (ROR-P PRS) by estrogen receptor status in the Pathways Study

(A) Kernel density plot of the ROR-P PRS stratified by estrogen receptor (ER) status. The ROR-P PRS was higher in ER-negative cancers than in ER-positive cancers, mean (standard deviation) of -30.5 (4.9) versus -31.1 (5.0) (t-statistic = -2.2, p = 0.03). (B) Forest plot of the associations between the ROR-P PRS and breast cancer-specific survival in ER-positive cancers (2,386 cases with 177 events) and ER-negative cancers (382 cases with 64 events). Cox proportional hazards models were adjusted for genetic ancestry (principal components 1-10). Hazard ratios per standard deviation are shown.

A.

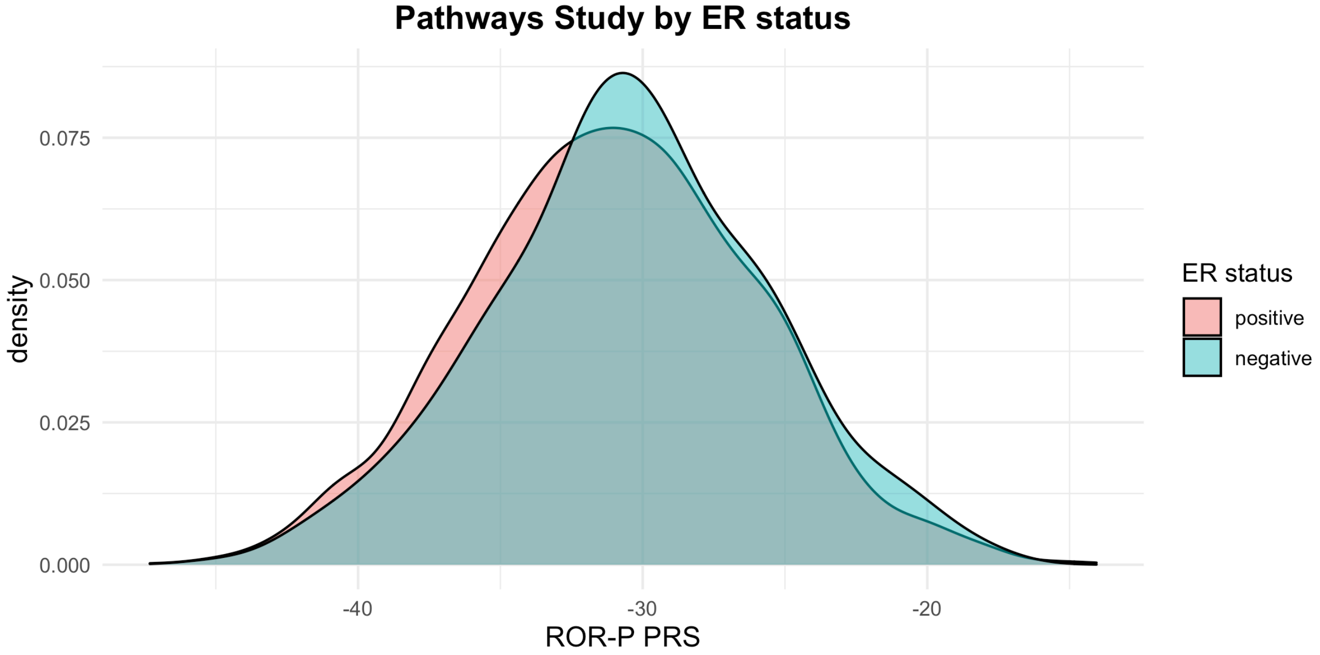

B.

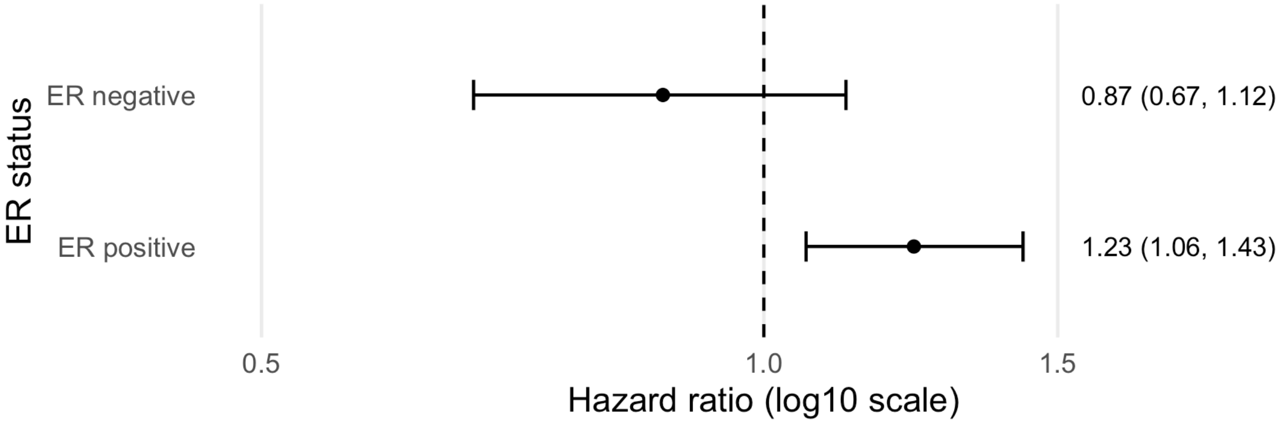

Supplement: Supplementary file 1 — Supplementary Materials [file 41698_2023_382_MOESM1_ESM.pdf]
