# Supplementary material for: Development and testing of a polygenic risk score for breast cancer aggressiveness
Source: NPJ Precis Oncol. 2023 May 15;7:42. doi: 10.1038/s41698-023-00382-z (PMC10185660; doi:10.1038/s41698-023-00382-z)
Supplement: Supplementary file 3 — REPORTING SUMMARY [file 41698_2023_382_MOESM3_ESM.pdf]

nature portfolio

Corresponding author(s): Yiwey Shieh

Last updated by author(s): Mar 27, 2023

Reporting Summary

Nature Portfolio wishes to improve the reproducibility of the work that we publish. This form provides structure for consistency and transparency in reporting. For further information on Nature Portfolio policies, see our Editorial Policies and the Editorial Policy Checklist.

Statistics

For all statistical analyses, confirm that the following items are present in the figure legend, table legend, main text, or Methods section.

n/a	Confirmed	
☐	☒	The exact sample size (*n*) for each experimental group/condition, given as a discrete number and unit of measurement
☐	☒	A statement on whether measurements were taken from distinct samples or whether the same sample was measured repeatedly
☐	☒	The statistical test(s) used AND whether they are one- or two-sided<br>*Only common tests should be described solely by name; describe more complex techniques in the Methods section.*
☐	☒	A description of all covariates tested
☐	☒	A description of any assumptions or corrections, such as tests of normality and adjustment for multiple comparisons
☐	☒	A full description of the statistical parameters including central tendency (e.g. means) or other basic estimates (e.g. regression coefficient) AND variation (e.g. standard deviation) or associated estimates of uncertainty (e.g. confidence intervals)
☐	☒	For null hypothesis testing, the test statistic (e.g. *F*, *t*, *r*) with confidence intervals, effect sizes, degrees of freedom and *P* value noted<br>*Give P values as exact values whenever suitable.*
☒	☐	For Bayesian analysis, information on the choice of priors and Markov chain Monte Carlo settings
☒	☐	For hierarchical and complex designs, identification of the appropriate level for tests and full reporting of outcomes
☐	☒	Estimates of effect sizes (e.g. Cohen's *d*, Pearson's *r*), indicating how they were calculated

Our web collection on statistics for biologists contains articles on many of the points above.

Software and code

Policy information about availability of computer code

Data collection	Plink (version 1.9) and VCFtools (v0.1.13) were used to extract genetic data.
Data analysis	Analyses were done using R version 4.1.2 (R Foundation, Vienna, Austria). Statistical code used to conduct the analyses can be found at https://github.com/shiehy/ROR-P_PRS.

For manuscripts utilizing custom algorithms or software that are central to the research but not yet described in published literature, software must be made available to editors and reviewers. We strongly encourage code deposition in a community repository (e.g. GitHub). See the Nature Portfolio guidelines for submitting code & software for further information.

Data

Policy information about availability of data

All manuscripts must include a data availability statement. This statement should provide the following information, where applicable:

- Accession codes, unique identifiers, or web links for publicly available datasets
- A description of any restrictions on data availability
- For clinical datasets or third party data, please ensure that the statement adheres to our policy

The Cancer Genome Atlas data are publicly available and were accessed through the Broad GDAC Firehose (https://gdac.broadinstitute.org/). METABRIC data are available through application to the METABRIC Data Access Committee and finalization of a Data Access Agreement. For this study, METABRIC data was accessed through the European Genome-Phenome Archive (https://ega-archive.org) under dataset IDs EGAD00010000266 (genotype) and EGAD00010000268 (gene

expression). I-SPY 2 data are available upon application to the I-SPY 2 TRIAL Data Access Committee and finalization of a Data Use Agreement. The UK Biobank data are available to approved researchers registered with the UK Biobank. The research was conducted with approved access to UK Biobank data under application number 14105. The Pathways Study genotype data are available on The database of Genotypes and Phenotypes (dbGaP) under study accession phs001534.v1.p1. Clinical and outcomes data are available upon application to the Pathways Study Steering Committee and require an IRB-approved collaboration.

Human research participants

Policy information about studies involving human research participants and Sex and Gender in Research.

Reporting on sex and gender	The analysis was restricted to individuals of self-reported female sex given that >99% of breast cancers occur in biological female sex.
Population characteristics	PRS development datasets (TCGA, METABRIC, I-SPY 2 TRIAL): Self-reported female sex, diagnosis of invasive breast cancer, available tumor gene expression and germline SNP genotyping data. Additional eligibility criteria for I-SPY 2 TRIAL can be found at https://clinicaltrials.gov/ct2/show/NCT01042379.<br><br>PRS validation datasets (UK Biobank, the Pathways Study): For UK Biobank, self-reported British White race, age 40-69 at time on enrollment (2006-2010), first diagnosis of invasive breast cancer after enrollment in UK Biobank, available germline SNP genotyping data. For Pathways Study, self-reported White race, age 21 and older with first diagnosis of invasive breast cancer 2006-2013, complete clinical and germline SNP genotype data.
Recruitment	PRS development datasets (TCGA, METABRIC, I-SPY 2 TRIAL): Specimens for TCGA were obtained at participating study sites in the United States of America. Specimens for the METABRIC Study were assembled from tumor banks in the United Kingdom and Canada. The I-SPY 2 TRIAL recruits at 36 locations in the United States of America, as documented here: https://clinicaltrials.gov/ct2/show/NCT01042379. The generalizability of these datasets is limited by several factors, such as recruitment site (academic centers), minimal size of tumor needed for adequate sampling/banking, intentional accounting for race/ethnicity (TCGA). I-SPY 2 is restricted to molecularly aggressive, locally advanced cancers.<br><br>PRS validation datasets (UK Biobank, the Pathways Study): UK Biobank is a population-based cohort that enrolled individuals aged 40-69 years across the UK between 2006-2010, with cancer diagnoses and deaths ascertained from national registries. The Pathways Study is a longitudinal cohort of women diagnosed with breast cancer at Kaiser Permanente Northern California. Participants included women aged 21 years and older with a first diagnosis of invasive breast cancer between 2006-2013.
Ethics oversight	The pooled analysis described in this manuscript was approved by the Biomedical Research Alliance of New York. The individual studies contributing data to this analysis each obtained approval from their respective ethical oversight committees.

Note that full information on the approval of the study protocol must also be provided in the manuscript.

Field-specific reporting

Please select the one below that is the best fit for your research. If you are not sure, read the appropriate sections before making your selection.

☒ Life sciences ☐ Behavioural & social sciences ☐ Ecological, evolutionary & environmental sciences

For a reference copy of the document with all sections, see nature.com/documents/nr-reporting-summary-flat.pdf

Life sciences study design

All studies must disclose on these points even when the disclosure is negative.

Sample size	PRS development datasets: total sample size was 2,363. This represented the maximum achievable based on our study criteria and overall limited availability of breast cancer datasets with tumor gene expression and germline SNP genotyping. These sample sizes are sufficient given that the goal of this analysis was not the discovery of novel genetic variants, but the re-fitting of the coefficients for previously discovered variants.<br><br>PRS validation datasets: sample size was 7,427 in UK Biobank and 2,769 in the Pathways Study. These sample sizes are the maximum achievable for our study criteria and appropriate for our objective of testing the performance of a polygenic risk score for breast cancer survival.
Data exclusions	PRS development datasets: We included all breast cancers from The Cancer Genome Atlas with the exception of samples corresponding to recurrent cancer (n=953). We included all cancers from the Molecular Taxonomy of Breast Cancer International Consortium (METABRIC) dataset with germline SNP and tumor gene expression data (n=496). We included cancers from the Investigation of Serial Studies to Predict Your Therapeutic Response with Imaging And molecular analysis 2 (I-SPY 2 TRIAL) that had undergone genotyping and for which gene expression data were available (n=914). For these three datasets, we excluded individuals with a missing call rate > 5%, based on minor allele frequency >0.5% and variant missing rate <5%.<br><br>PRS validation datasets: In the UK Biobank, we included women of Self-reported British White race with incident breast cancer (n=7,427),

	defined as an invasive breast cancer diagnosis occurring on a date later than the date of enrollment in UK Biobank. We excluded individuals with a variant missing rate of >3% based on minor allele frequency >0.01 and heterozygosity less than 5 standard deviations from the mean. In the Pathways Study, we included 2,769 women with self-reported White race. We excluded individuals based on discordance between duplicate samples.
Replication	We used two independent datasets, the UK Biobank and the Pathways Study, to examine the association between our novel polygenic risk score (PRS) and survival. We reported the results in each individual dataset as well as that of a meta-analysis including both datasets. For replication of the PRS in other datasets, we have included the variants and effect sizes in our supplemental data, as well as a description of the methods used to calculate it.
Randomization	Randomization was not relevant to this study since it did not involve allocation of experimental groups to a specific intervention.
Blinding	Blinding was not relevant to this study. Measures and outcomes were already ascertained, and ascertainment of measures and outcomes was done independently from each other.

Reporting for specific materials, systems and methods

We require information from authors about some types of materials, experimental systems and methods used in many studies. Here, indicate whether each material, system or method listed is relevant to your study. If you are not sure if a list item applies to your research, read the appropriate section before selecting a response.

Materials & experimental systems

n/a	Involved in the study	
☒	☐	Antibodies
☒	☐	Eukaryotic cell lines
☒	☐	Palaeontology and archaeology
☒	☐	Animals and other organisms
☒	☐	Clinical data
☒	☐	Dual use research of concern

Methods

n/a	Involved in the study	
☒	☐	ChIP-seq
☒	☐	Flow cytometry
☒	☐	MRI-based neuroimaging
